# Variable-Parameter Impedance Control of Manipulator Based on RBFNN and Gradient Descent

**DOI:** 10.3390/s25010049

**Published:** 2024-12-25

**Authors:** Linshen Li, Fan Wang, Huilin Tang, Yanbing Liang

**Affiliations:** 1 Xi’an Institute of Optics and Precision Mechanics of CAS, Xi’an 710119, China; lilinshen2022@opt.ac.cn (L.L.); wangfan@opt.ac.cn (F.W.); tanghuilin@opt.ac.cn (H.T.); 2 School of Optoelectronics, University of Chinese Academy of Sciences, Beijing 100049, China; 3 Key Laboratory of Space Precision Measurement Technology of CAS, Xi’an 710119, China

**Keywords:** manipulator, impedance control, gradient descent, RBFNN

## Abstract

During the interaction process of a manipulator executing a grasping task, to ensure no damage to the object, accurate force and position control of the manipulator’s end-effector must be concurrently implemented. To address the computationally intensive nature of current hybrid force/position control methods, a variable-parameter impedance control method for manipulators, utilizing a gradient descent method and Radial Basis Function Neural Network (RBFNN), is proposed. This method employs a position-based impedance control structure that integrates iterative learning control principles with a gradient descent method to dynamically adjust impedance parameters. Firstly, a sliding mode controller is designed for position control to mitigate uncertainties, including friction and unknown perturbations within the manipulator system. Secondly, the RBFNN, known for its nonlinear fitting capabilities, is employed to identify the system throughout the iterative process. Lastly, a gradient descent method adjusts the impedance parameters iteratively. Through simulation and experimentation, the efficacy of the proposed method in achieving precise force and position control is confirmed. Compared to traditional impedance control, manual adjustment of impedance parameters is unnecessary, and the method can adapt to tasks involving objects of varying stiffness, highlighting its superiority.

## 1. Introduction

In applications such as polishing, gripping, and human–machine interaction, a manipulator needs to maintain a certain contact force in addition to stable tracking of position signal. This requires the implementation of compliance control of the manipulator. Compliance control can be categorized into passive and active versions [1]. The former achieves the compliance of systems by relying on mechanically structured hardware. Therefore, such a control method has limitations in control accuracy and environmental adaptability. The latter can be segmented into hybrid force/position control and impedance control. A hybrid force/position control system consists of a position controller and a force controller. With this method, the task space of the manipulator is divided into position subspace and force subspace, and controller switching is achieved through a diagonal array S. This approach involves a large computational load, leading to a delay in control switching [2,3,4,5,6]. In contrast, impedance control is an approach to control force and position simultaneously by regulating the dynamic relation between the contact force and the motion state, decreasing the computational load. In addition, it is easier to implement.

Proposed by Hogan in 1984 [7], impedance control describes the transfer relationship between contact force and position through an impedance analogy based on the concept and characteristics of impedance in electrical circuits. It has a broad range of applications, including manipulator position control, manipulator force control, human–machine collaboration, and multi-manipulator collaboration. Multi-priority, multi-objective control for trajectory tracking, proposed by A. Dietrich and C. Ott, is to ensure that the end-effector maintains a fixed impedance during the tracking of a position signal [8]. Force tracking impedance control based on higher-order sliding mode control, proposed by H. Khan et al., eliminates the jitter in force control, reducing the force tracking error compared to the conventional sliding mode controller [9]. The simplified human arm impedance model, built by R. Wu et al., fits to human operating habits and enhances the robot’s learning ability to carry out human–robot interaction tasks in variable impedance conditions [10]. T. Wimbock et al. defined the impedance relationships between two manipulators and between manipulators and objects at different task stages. By integrating these concepts, they designed an impedance controller for a humanoid two-handed robot to enhance two-handed cooperation for tasks, for example, unscrewing cans [11].

In recent years, experts and scholars have integrated impedance control with intelligent control methods. For example, Li, D.-Y. et al. developed an adaptive sliding mode controller based on time delay estimation by combining impedance control with fuzzy control. This controller can simplify the manipulator dynamics model through time delay estimation and eliminate the jitter in the sliding mode controller with an adaptive approach [12]. X. Wang et al. combined impedance control with neural networks to improve position tracking by means of adaptive neural networks [13]. Z. Li et al. designed a dual closed-loop controller that uses reinforcement learning to optimize the proposed linear quadratic regulator by using impedance control together with reinforcement learning. This allows for stable tracking of the trajectory and more stable control of the interaction force [14]. X. Liang et al. combined impedance control with an iterative learning control method to correct position errors and improve position control accuracy [15]. Patino, J. et al. introduced a novel machine learning-based methodology for predicting the damping ratio in redundant Cartesian impedance-controlled robots [16]. They leveraged extensive experimental data to estimate the damping ratio, thereby eliminating the reliance on complex mathematical equations or physical models, which enhances prediction accuracy and model generalization.

In applications where a manipulator is used to perform gripping tasks [17], there are always changes in the details about the contact between the manipulator and the environment, such as the precise position, stiffness, material, and shape of the object in contact. When contact operations are performed using conventional fixed impedance control, the impedance parameters need to be adjusted manually in different contact environments so that the system responds in a desired manner. For this reason, the controller should be designed to automatically adjust the impedance parameters according to the changing contact conditions. Gan, Y.-H. et al. developed an adaptive method to adjust the damping parameters online through combining impedance control with an adaptive method. This method can fully track the desired force signal at the cost of positional steady-state error [18]. Zheng S. et al. proposed an adaptive impedance control algorithm in which online tuning is combined with offline optimization to allow for the online tuning of the stiffness parameter k with an adaptive algorithm and the offline optimization of the parameters m and b via neural networks in case of contact force changes [19]. W. Lu et al. proposed a differential-free impedance control method for variable stiffness. This method can be used to derive the relationship between the contact force and the contact surface stiffness and position based on the impedance relationship, enabling the adjustment of stiffness parameters, but it is only applicable to scenarios where position signals are constant [20]. L. Penco et al. proposed a method for automatically learning the controller configuration of redundant robots [21]. They employ multi-objective optimization to identify Pareto-optimal solutions that balance performance and robustness, thereby enabling the tracking of various desired task trajectories while ensuring the robot’s balance.

In conclusion, these methods can achieve parameter adaptation under certain conditions at the cost of steady-state errors or offline optimization. To address such a problem, this paper proposed a variable-parameter impedance control method based on gradient descent and RBFNN. This method can reduce the position and force steady-state errors while allowing for online adjustment of impedance parameters. Moreover, a position-based impedance control structure was used. In this structure, the inner loop is a sliding mode controller for performing position control, eliminating the impact of the uncertainties in dynamics modeling and keeping accurate position control; the outer loop is an impedance controller, which is based on a gradient descent method and iterative learning control to achieve online adjustment of impedance parameters. Simulations and experiments verified the effectiveness of the method in enhancing simultaneous and accurate position and force control. The main contributions are as follows:

1. A variable-parameter impedance control method was proposed. It integrated RBFNN with the gradient descent method to address the challenge of gripping different objects with varying stiffness, allowing for the online adjustment of impedance parameters.

2. A sliding mode controller was developed to reduce the impact of the uncertainties in the dynamics modeling of the manipulator on the control outcomes. This enhanced the accuracy of the inner-loop position control by compensating for unknown dynamics.

## 2. Modeling of Manipulator Dynamics

The dynamics of the n-jointed manipulator in the joint space are given by:

(1) $$M_{J}\ddot{q}+C_{J}\left(q,\dot{q}\right)\dot{q}+G_{J}\left(q\right)=\tau +\tau_{e}$$

where $q\in R^{n}$, $\dot{q}\in R^{n}$ and $\ddot{q}\in R^{n}$ are the vector of joint angles, velocities, accelerations, respectively; $M_{J}\in R^{n\times n}$ is a symmetric positive-definite matrix that is configuration-dependent and depends on the positions of the links and joints, which in turn depend on the joint angles q; $C_{J}\left(q,\dot{q}\right)\dot{q}\in R^{n}$ is the centrifugal and the Coriolis force term; $G_{J}\left(q\right)\in R^{n}$ is the gravitational term; $\tau \in R^{n}$ is the driving force; $\tau_{e}\in R^{n}$ is the force in contact with the environment.

Since the relationship between joint angular velocity and the Cartesian space velocity is as follows:

(2) $$\dot{x}=J\dot{q}$$

Therefore, the dynamics equation can also be described as:

(3) $$M\ddot{x}+C\left(x,\dot{x}\right)\dot{x}+G\left(x\right)=f+f_{e}$$

where $x\in R^{n}$, $\dot{x}\in R^{n}$, $\ddot{x}\in R^{n}$ are the position vector, velocity vector, and acceleration vector in the Cartesian space; $M\in R^{n\times n}$ is the inertia matrix; $C\left(x,\dot{x}\right)\dot{x}\in R^{n}$ is the centrifugal and the Coriolis force term; $G\left(x\right)\in R^{n}$ is the gravitational term; $f\in R^{n}$ is the driving force; $f_{e}\in R^{n}$ is the force in contact with the environment.

The moment a manipulator end-effector makes contact with an object, it can be viewed as a collision. Therefore, the contact force can be estimated using a collision model. A linear spring-damping model (Kelvin-Voigt model) was proposed by Kelvin and Voigt in 1960 [22]:

(4) $$F=K\delta +D\dot{\delta }$$

where K is the constant coefficient stiffness, D is the constant coefficient damping, δ is the contact deformation variable, and $\dot{\delta }\;$ is the relative collision velocity. Since the application scenario of this paper involves using a manipulator to grasp an object, the second term (the damping term) can be ignored because the end velocity of the manipulator is very small during the grasping process. 

## 3. Controller Design

### 3.1. Introduction to Impedance Controllers

Impedance control establishes a relationship between a contact force and a position, known as the impedance relationship. By adjusting the impedance parameters, the system can achieve a steady state with zero steady-state error. The impedance relationship is typically expressed as a second-order equation, which works equally in joint space and task space:

(5) $$M\Delta \ddot{x}+B\Delta \dot{x}+K\Delta x=\Delta f$$

(6) $$\Delta x=x_{d}-x_{e}$$

(7) $$\Delta f=f_{d}-f_{e}$$

where $M\in R^{n\times n}$, $B\in R^{n\times n}$**,** and $K\in R^{n\times n}$ represent the inertia parameter, damping parameter, and stiffness parameter in the impedance relation, respectively, all of which are positive definite matrices; Δx, $\Delta \dot{x}$, $\Delta \ddot{x}$ represent the position, velocity, and acceleration error vectors, respectively; Δf is the force error vector of the end-effector; $x_{d}$ and $x_{e}$ are the desired and actual position vectors of the end-effector, respectively; $f_{d}$ and $f_{e}$ are the desired and actual force vectors for the end-effector in contact with the environment.

**Figure 1:**
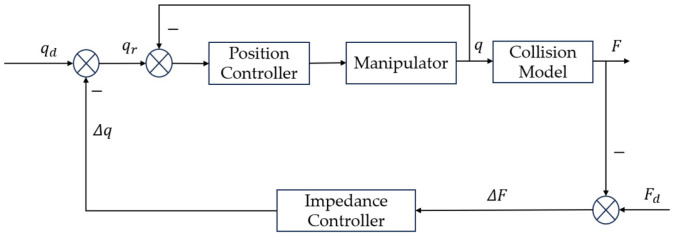
The position-based impedance control structure.

Impedance controllers are typically categorized into two types: position-based impedance control structure and force-based impedance control structure [23]. The position-based impedance control structure is adopted in this paper. Its control block diagram is shown in Figure 1; we can conceptualize this as a dual closed-loop control architecture. The inner loop comprises the position controller, ensuring rapid and stable tracking of the desired position signal by the manipulator. The outer loop includes the impedance controller, which transforms the external force applied to the end-effector into a position correction value. The desired position signal is adjusted using this position correction value and then fed into the position controller. This structure adheres to the principles defined for each control law without any violations.

### 3.2. Impedance Controller Analysis

#### 3.2.1. Effect of Impedance Parameters on System Performance

Given that the manipulator is a highly nonlinear and coupled system, incorporating nonlinear control methods such as sliding mode control can simplify the design and analysis of the control strategy by linearizing its behavior under specific operating conditions. By designing controllers for each joint, the n-dimensional system can be decoupled into single-degree-of-freedom subsystems. To facilitate analysis and verification, the discussions in this section are based on a single-joint model in joint space, with its impedance model expressed as follows:

(8) $$m\Delta \ddot{x}+b\Delta \dot{x}+k\Delta x=\Delta f$$

where Δx is the displacement error of the joint, and Δf is the force error on the joint. The Laplace transform of Equation (8):

(9) $$\Delta F\left(s\right)=\left(ms^{2}+bs+k\right)\Delta X$$

For Equation (9), both sides are simultaneously divided by ΔX, yielding the transfer function of a second-order system:

(10) $$\frac{\Delta X}{\Delta F}=\frac{1}{ms^{2}+bs+k}=\frac{\frac{1}{m}}{s^{2}+\frac{b}{m}s+\frac{k}{m}}=\frac{\frac{1}{m}}{s^{2}+2\xi \omega_{n}s+\omega_{n}^{2}}$$

According to the Routh–Hurwitz stability criterion, when m > 0, b > 0, and k > 0, the second-order system in Equation (10) is stabilized. The relationship between the damping ratio ξ, the undamped natural frequency $w_{n}$, and the three impedance parameters can be derived from the above equation:

(11) $$\xi =\frac{b}{2\sqrt{km}\;}$$

(12) $$\omega_{n}=\sqrt{\frac{k}{m}}$$

Assuming that the system is underdamped, the relationship between the impedance parameters can be:

(13) $$b<2\sqrt{km}$$

#### 3.2.2. Force Steady-State Error

The following assumptions are made:

(1) The effect of inner loop position control on the system is ignored;
(2) When the end-effector of the manipulator is at $x^{0}$ the contact force on the surface of the object is zero;
(3) The contact stiffness of the object is $k_{e}$.

From Equation (9), we can obtain:

(14) $$x_{d}-x=\frac{1}{{ms}^{2}+bs+k}\Delta f$$

According to Equations (4) and (14), the error between the actual and desired contact force of the end-effector can be described as:

(15) $$\begin{array}{cc} \Delta f & =f_{d}-k_{e}\left(x_{0}-x\right)\\ & =f_{d}-k_{e}x_{0}+k_{e}\left(x_{d}-\frac{1}{{ms}^{2}+bs+k}\Delta f\right) \end{array}$$

and derived as follows:

(16) $$\Delta f=\frac{{ms}^{2}+bs+k}{{ms}^{2}+bs+k+k_{e}}\left[f_{d}-k_{e}\left(x_{0}-x_{d}\right)\right]$$

According to the final-value theorem, we obtain the force steady state error $\Delta f_{ss}$:

(17) $$\Delta f_{ss}=\frac{k}{k+k_{e}}\left[f_{d}-k_{e}\left(x_{0}-x_{d}\right)\right]$$

Therefore, for the force steady state error to be zero, the desired position, the desired force and the environmental stiffness need to satisfy the relational equation:

(18) $$x_{d}=x_{0}-\frac{f_{d}}{k_{e}}$$

### 3.3. Inner Loop Position Controller Design

In this section, we focus on designing a position controller for the inner loop of the position-based impedance control architecture.

The manipulator system is inherently nonlinear, and the friction in the manipulator joints, along with unknown external perturbations, is difficult to model accurately in practice. These hard-to-model components and nonlinear characteristics can be considered as perturbations affecting the system. Sliding Mode Control (SMC) is a robust nonlinear control technique that is extensively utilized for trajectory tracking in manipulators. Consequently, employing SMC to mitigate these perturbations enhances the stability and accuracy of the system.

The discussion in this section focuses on the joint space. To illustrate the resistance of SMC to the aforementioned perturbations, we consider a multi-jointed manipulator. Nonetheless, the subsequent conclusions are equally applicable to single-joint systems. Let q denote the actual joint position and $q_{d}$ represent the desired joint position. The error signal, which serves as the input to the SMC, is defined as:

(19) $$e=q_{d}-q$$

and can be further defined as:

(20) $$\dot{q_{r}}=\dot{q_{d}}+\Lambda \left(q_{d}-q\right)$$

where Λ is a positive diagonal matrix. Assuming the existence of a vector $P\in R^{m}$, the dynamics model has the following dynamics properties:

(21) $$H\left(q\right)\ddot{q_{r}}+C\left(q,\dot{q}\right)\dot{q_{r}}+G\left(q\right)=Y\left(q,\dot{q},\dot{q_{r}},{\ddot{q}}_{r}\right)P$$

(22) $$\tilde{H}\left(q\right)\ddot{q_{r}}+\tilde{C}\left(q,\dot{q}\right)\dot{q_{r}}+\tilde{G}\left(q\right)=Y\left(q,\dot{q},\dot{q_{r}},{\ddot{q}}_{r}\right)\tilde{P}$$

where Equation (21) is the real manipulator dynamics properties, and Equation (22) is the error between the real properties and the estimated properties.

Defining Sliding Mold Surfaces **s**

(23) $$s=\dot{q_{r}}-\dot{q}=\left(\dot{q_{d}}-\dot{q}\right)+\Lambda \left(q_{d}-q\right)=\dot{e}+\Lambda e$$

Defining the Lyapunov function

(24) $$V\left(t\right)=\frac{1}{2}s^{T}H\left(q\right)s$$

Differentiating Equation (24) with respect to time results in

(25) $$\begin{array}{cc} \dot{V}\left(t\right) & =s^{T}H\left(q\right)\dot{s}+\frac{1}{2}s^{T}\dot{H}\left(q\right)s\\ & =s^{T}H\left(q\right)\dot{s}+s^{T}C\left(q,\dot{q}\right)s\\ & =s^{T}\left[H\left(q\right)\left(\ddot{q_{r}}-\ddot{q}\right)+C\left(q,\dot{q}\right)\left(\dot{q_{r}}-\dot{q}\right)\right]\\ & =s^{T}\left[H\left(q\right)\ddot{q_{r}}+C\left(q,\dot{q}\right)\ddot{q_{r}}+G\left(q\right)-\tau \right] \end{array}$$

The control law is designed as:

(26) $$\tau =\hat{H}\left(q\right)\ddot{q_{r}}+\hat{C}\left(q,\dot{q}\right)\dot{q_{r}}+\hat{G}\left(q\right)+\tau_{s}$$

where $\hat{H}\left(q\right)\ddot{q_{r}}+\hat{C}\left(q,\dot{q}\right)\dot{q_{r}}+\hat{G}\left(q\right)$ represent the estimated dynamics properties, and the $\tau_{s}$ needs to be designed.

We determined an expression for $\tau_{s}$ such that $\dot{V}\left(t\right)\leq 0$. Substituting Equation (26) into Equation (25), we have:

(27) $$\begin{array}{cc} \dot{V}\left(t\right) & =s^{T}\left[H\ddot{q_{r}}+C\ddot{q_{r}}+G-\hat{H}\ddot{q_{r}}-\hat{C}\dot{q_{r}}-\hat{G}-\tau_{s}\right]\\ & =s^{T}\left[\tilde{H}\ddot{q_{r}}+\tilde{C}\ddot{q_{r}}+\tilde{G}-\tau_{s}\right]=s^{T}\left[Y\tilde{P}-\tau_{s}\right] \end{array}$$

where $\tilde{P}={\left[{\tilde{P}}_{1},\ldots {\tilde{P}}_{10n}\right]}^{T}$, $\left|{\tilde{P}}_{i}\right|<a_{j}$, i=1,…,10n

$Y=[Y_{ij}^{r}]$, $\left|Y_{ij}^{r}\right|<{\bar{Y}}_{ij}^{r}$, i=1,…,n, j=1,…,10n

If $\tau_{s}$ is:

(28) $$\tau_{s}=ksgn\left(s\right)+s$$

where $k={\left[k_{1},\ldots ,k_{n}\right]}^{T}$, $k_{i}=\sum_{j=1}^{10n}{\bar{Y}}_{ij}^{r}a_{j}$, i=1,…,n

We can obtain:

(29) $$\dot{V}\left(t\right)=s^{T}\left[Y\tilde{P}-\tau_{s}\right]\leq -\sum_{i=1}^{n}s_{i}^{2}\leq 0$$

### 3.4. Variable Parameters Impedance Controller Design

**Figure 2:**
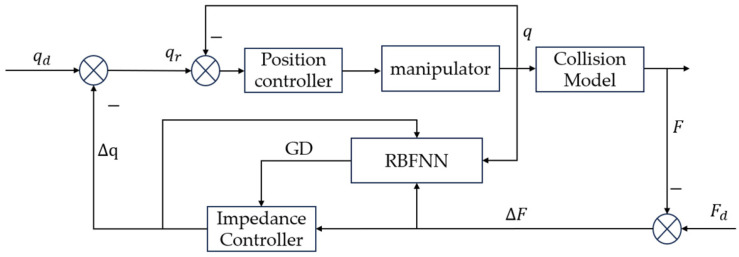
Structure of the variable-parameter impedance controller.

In manipulator grasping tasks, varying object stiffness highlights the inadequacy of impedance controllers with constant parameters. This paper proposes a variable-parameter impedance control method based on gradient descent (GD) and radial basis function neural network (RBFNN), with the control system block diagram shown in Figure 2. The process consists of two key stages: system identification and self-adjustment of impedance parameters. Unlike traditional neural network adaptive control, this method eliminates the need for offline training. The impedance parameters are tuned using gradient descent (GD), a method that requires an accurate mathematical model of the system. Given the difficulty in achieving an accurate model due to various perturbations, the system identification in joint space is performed using a neural network.

RBFNN possess robust input-output mapping capabilities and are highly computationally efficient. Thus, an RBFNN is selected for system identification. System identification is performed using the system’s inputs and outputs, according to the following expression [24], with l=1 and m=2 in this study.

(30) $$\hat{y}\left(n+1\right)=NN\left[y\left(n\right),\ldots y\left(n-l+1\right),u\left(n\right),\ldots u\left(n-m+1\right)\right]$$

where y represents the output of the system at the first *n* − *l* + 1 to the first n moments, u represents the input of the system at the first *n* − *m* + 1 to the first n moments, and $\hat{y}\left(n+1\right)$ represents the estimated output of the system by RBFNN at the n + 1 moment.

**Figure 3:**
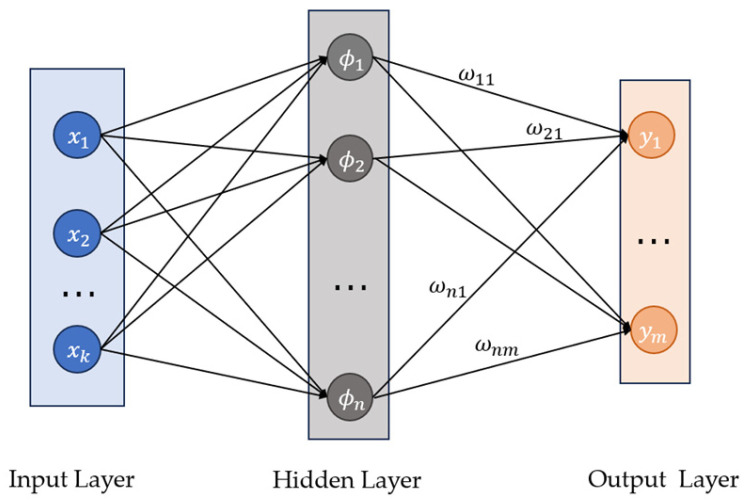
Structure diagram of the RBFNN.

The structure of the three-layer RBFNN is depicted in Figure 3, consisting of an input layer, a hidden layer, and an output layer [25,26,27,28]. In the hidden layer, the activation function used is a Gaussian function.

(31) $$\phi_{i}=e^{-\frac{{\left\|x-c_{i}\right\|}^{2}}{2b_{i}^{2}}}$$

where $c_{i}$ represents the center of node i and $b_{i}$ represents the width of node i. After passing through the activation function, the input variables are weighted, summed, and combined to produce the final output:

(32) $$y_{j}=\sum_{i=1}^{n}\omega_{ij}\phi_{i}$$

where $\omega_{ij}$ represents the weight from the hidden layer node i to the output layer node j and $\phi_{i}$ is a Gaussian function, which is expressed in Equation (31). From Equations (31) and (32), it can be deduced that three sets of parameters are adjustable within the RBFNN.

Define the error metric function for the RBFNN approximation as:

(33) $$J_{1}=\frac{1}{2}{\left(y-y_{m}\right)}^{2}$$

where y represents the actual system output, and $y_{m}$ denotes the neural network output. If the performance metrics do not meet expectations after these calculations, the neural network undergoes data updates. The three sets of neural network parameters are updated using the gradient descent method [29,30]:

(34) $$\begin{array}{cc} \omega_{ij}\left(n\right) & =\omega_{ij}\left(n-1\right)+\eta \left(y-y_{m}\right)\frac{\partial y\left(n-1\right)}{\partial w_{ij}\left(n-1\right)}\\ & +\alpha \left[\omega_{ij}\left(n-1\right)-\omega_{ij}\left(n-2\right)\right] \end{array}$$

(35) $$\begin{array}{cc} c_{j}\left(n\right) & =c_{j}\left(n-1\right)+\eta \left(y-y_{m}\right)\frac{\partial y\left(n-1\right)}{\partial c_{j}\left(n-1\right)}\\ & +\alpha \left[c_{j}\left(n-1\right)-c_{j}\left(n-2\right)\right] \end{array}$$

(36) $$\begin{array}{cc} b_{j}\left(n\right) & =b_{j}\left(n-1\right)+\eta \left(y-y_{m}\right)\frac{\partial y\left(n-1\right)}{\partial b_{j}\left(n-1\right)}\\ & +\alpha \left[b_{j}\left(n-1\right)-b_{j}\left(n-2\right)\right] \end{array}$$

where η represents the learning rate, α denotes the momentum factor and n is the iteration number.

To ensure that the system output force accurately tracks the desired force, a performance metric function is introduced, the following discussion focuses on a single-joint system within the joint space:

(37) $$J_{2}=\frac{1}{2}{\left(f_{d}-f_{e}\right)}^{2}$$

The partial derivative of $f_{e}$ is:

(38) $$\frac{\partial f_{e}(n)}{\partial \Delta X\left(n\right)}\approx \frac{\partial {k_{e}y}_{m}\left(n\right)}{\partial \Delta X\left(n\right)}=\sum_{j=1}^{m}{k_{e}\omega }_{j}\phi_{j}\frac{c_{j}-x\left(n\right)}{b_{j}^{3}}$$

Based on Equation (38), the impedance parameters (*b*, *k*) can be adjusted online using the gradient descent method:

(39) $$\Delta b=-\eta \left[y_{imp}\left(n\right)-y\left(n\right)\right]\frac{\partial y\left(n\right)}{\partial \Delta X\left(n\right)}\frac{\partial \Delta X\left(n\right)}{\partial b}$$

(40) $$\Delta k=-\eta \left[y_{imp}\left(n\right)-y\left(n\right)\right]\frac{\partial y\left(n\right)}{\partial \Delta X\left(n\right)}\frac{\partial \Delta X\left(n\right)}{\partial k}$$

(41) $$b=b_{0}+\Delta b$$

(42) $$k=k_{0}+\Delta k$$

where $b_{0}$ and $k_{0}$ in the above equation represent initial values determined empirically and through theoretical derivations, and *n* is the iteration number. Since the closed-loop system is a second-order system, the system can be stabilized as long as the impedance parameters are guaranteed to be positive during the tuning process.

## 4. Simulation Verification

**Figure 4:**
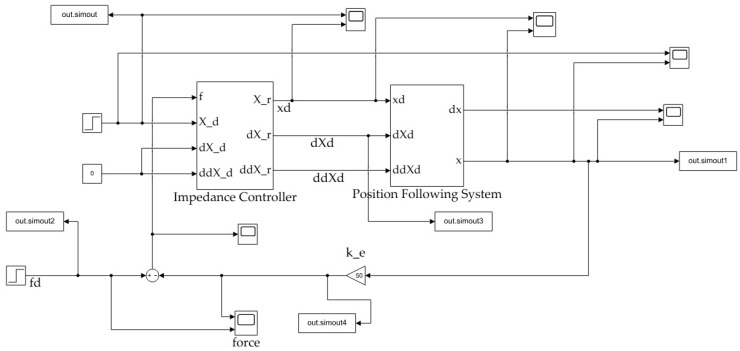
Schematic structure of the impedance control system simulation.

To evaluate the effectiveness of the adaptive impedance control method based on RBF neural networks simulation analysis was performed using the MATLAB 2021a/Simulink platform, comparing it to constant impedance control. The simulation schematic, depicted in Figure 4, integrates a closed-loop sliding mode controller within the position control framework, with the impedance controllers differing between the two scenarios. The controlled object, the manipulator’s end-effector (gripper), is modeled as a single motor-linkage system, with the assumption that friction acts as viscous resistance. The simulations in this section are conducted within the joint space as well. The key parameters are defined as follows: rotational moment of inertia $M=0.5\;kg\cdot m^{2}$, linkage mass m=1 kg, linkage length r=0.1 m, viscous resistance coefficient µ=0.1 N·s/m, and gravitational acceleration $g=9.8\;m/s^{2}$. The parameters of the SMC for position control are set as Λ=10, *k* = 5. Note that the parameter k here refers to the gain applied to the sign function in the sliding mode control law, intended to counteract disturbances in the system. This k should not be confused with the stiffness coefficient k used in the impedance controller.

### 4.1. Simulation Verification of Constant Impedance Control System

**Figure 5:**
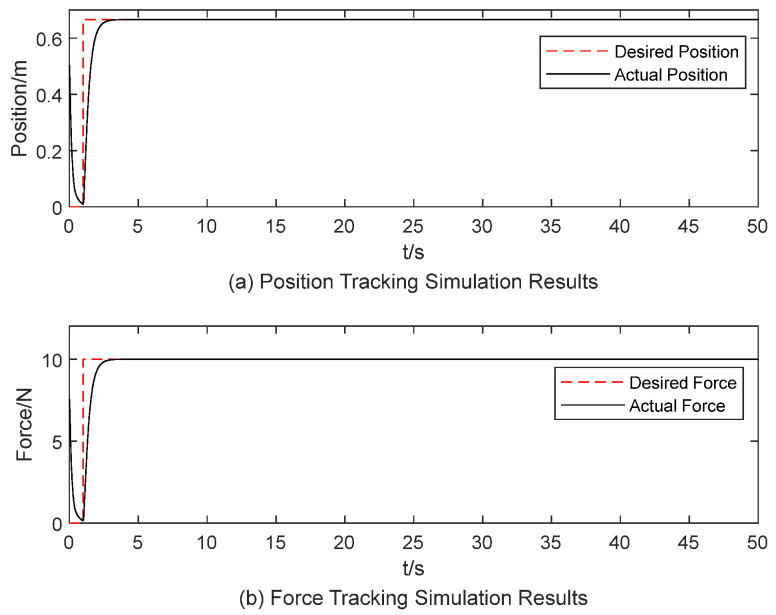
Simulation results of constant impedance parameters for an object with stiffness of 15 N/m.

**Figure 6:**
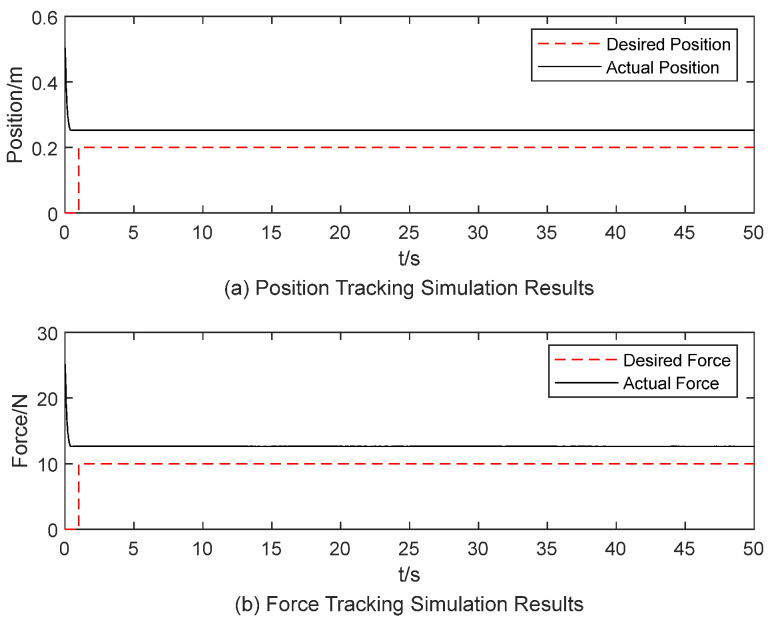
Simulation results of constant impedance parameters for an object with stiffness of 50 N/m.

According to the conclusions drawn in Section 3.2, a set of parameters were manually configured: m=1, b=15, k=50, $f_{d}=10\;N$, $x_{d}=0.666\;m$. For an object stiffness of 15 N/m, the end-effector’s reference position and desired force tracking results are shown in Figure 5. The results indicate a steady-state error of zero, no overshoot, and a settling time of 3 s. When the object stiffness is increased to 50 N/m and the desired position adjusted to $x_{d}=0.2\;m$, the corresponding tracking results for the end-effector’s reference position and desired force are shown in Figure 6. In Figure 6, the simulation results show steady-state error exists. These results demonstrate that constant impedance parameters are unsuitable for varying contact stiffness conditions.

### 4.2. Simulation Validation of the Variable-Parameter Impedance Control Method

In this section, the mass parameter m=1 is held constant, while the damping b and stiffness *k* are adjusted adaptively. Based on the findings in Section 3.2, the desired force is set to 10 N, the desired position to 0.666 m, and the initial values of the adaptive impedance parameters are b=4 and k=5. The neural network parameters are configured as shown in Table 1.

**Figure 7:**
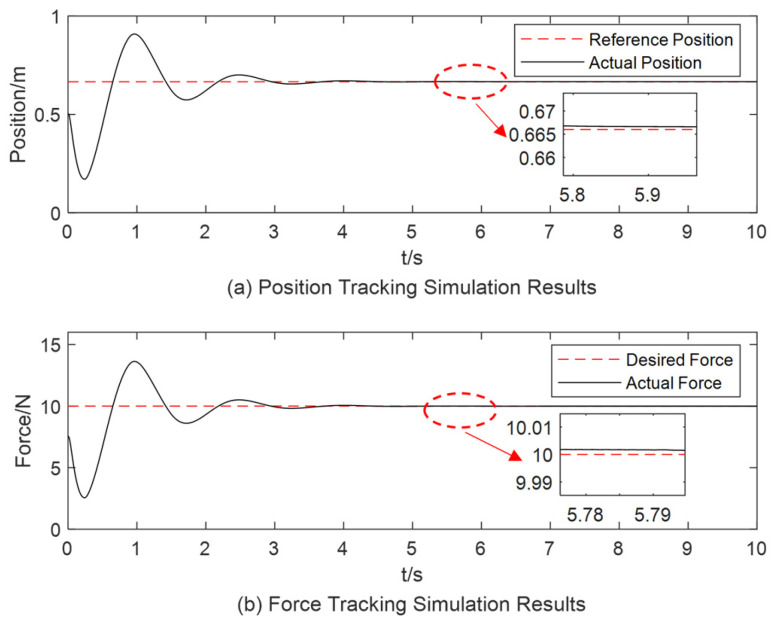
Simulation results for object stiffness of 15 N/m using the variable-parameter impedance controller.

**Figure 8:**
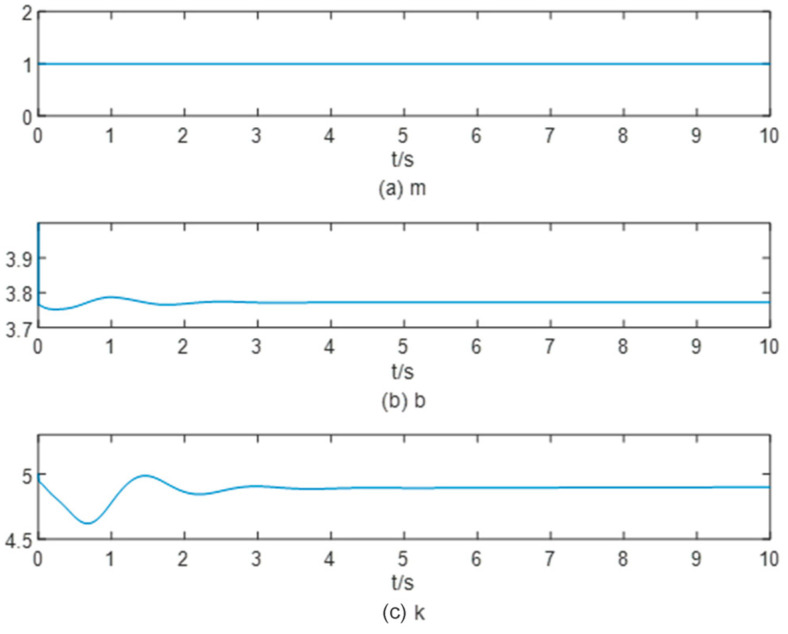
The change process of the impedance parameter when the object stiffness is 15 N/m.

At an object stiffness of 15 N/m, the end-effector’s reference position and desired force tracking results, presented in Figure 7, show the system’s precise tracking capabilities. The trend of impedance parameters during the whole process is shown in Figure 8. In Figure 7, we can observe that the system produces a 20% overshoot when it starts to track the desired position and the desired force signal, then the system error gradually narrows down. At around 4 s, the steady state error approaches zero. In Figure 8, the stiffness factor k and the damping factor *b* adjusted from b=4 and k=5,stabilized at b=3.78, k=4.85.

**Figure 9:**
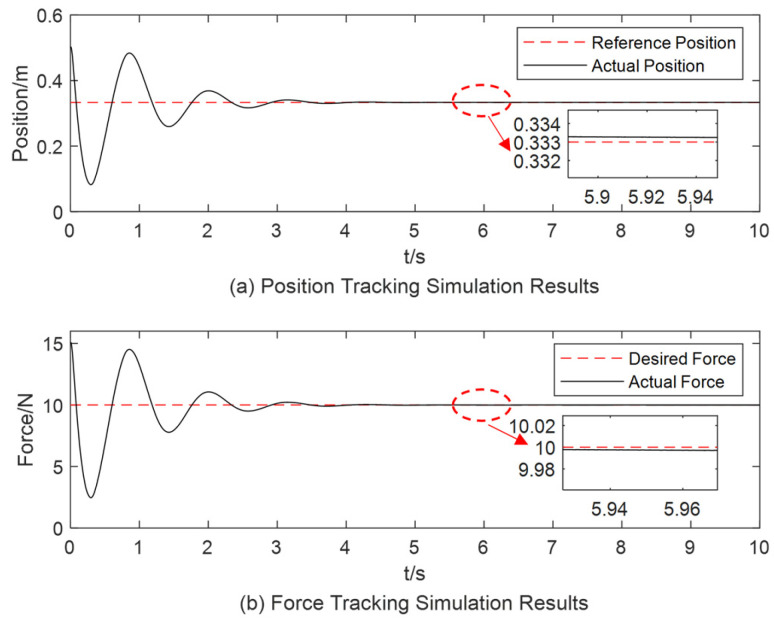
Simulation results for object stiffness of 30 N/m using the variable-parameter impedance controller.

**Figure 10:**
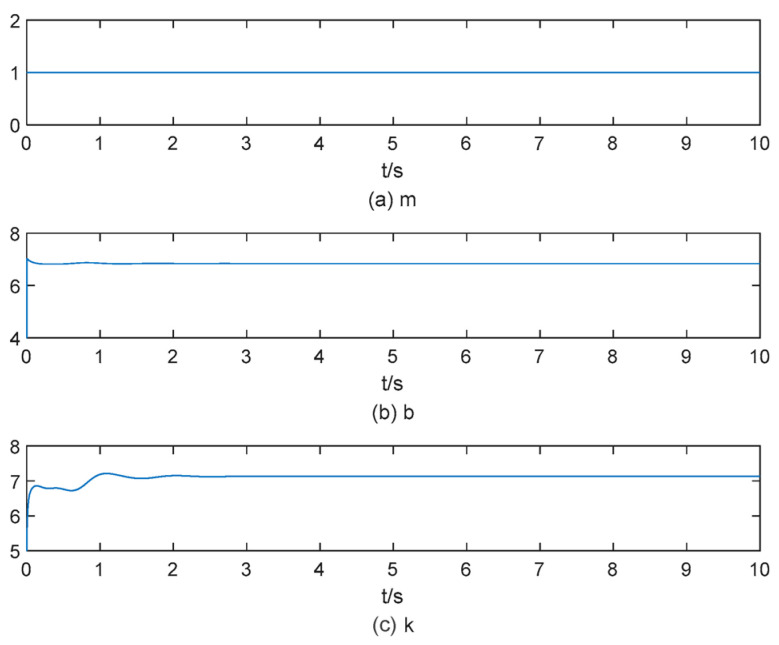
The change process of the impedance parameter when the object stiffness is 30 N/m.

As environmental stiffness changes, based on conclusions drawn in Section 3.2, the position signal must be adjusted to eliminate the force steady-state error. Therefore, when the object stiffness is increased to 30 N/m and the position signal is adjusted to $x_{d}=0.333\;m$, affecting the end-effector’s reference position and desired force tracking results, as depicted in Figure 9. The variation in impedance parameters is illustrated in Figure 10. In Figure 9, there is still a 50% overshoot; furthermore, the position and force steady-state errors converge to zero around *t* = 4 s. In Figure 10, the impedance parameters stabilize at b=6 and k=6.2 at around 4.5 s. 

**Figure 11:**
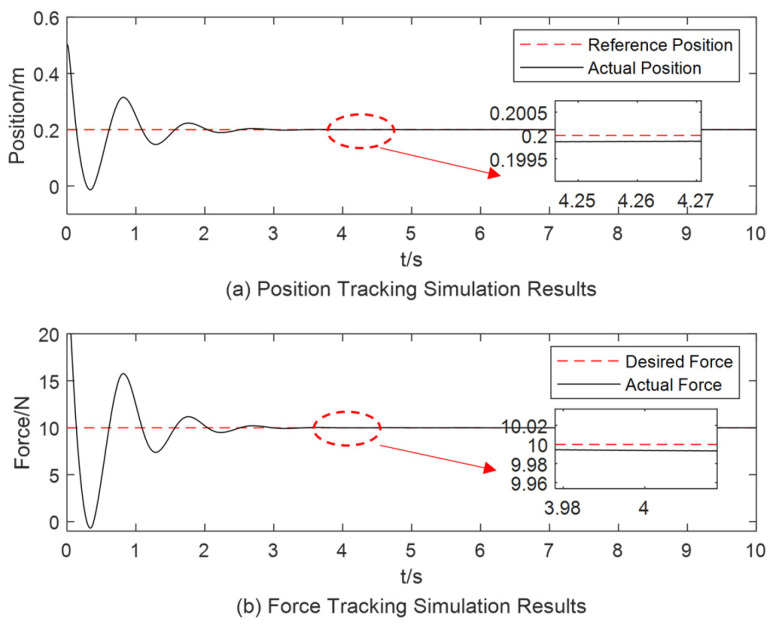
Simulation results for object stiffness of 50 N/m using the variable-parameter impedance controller.

**Figure 12:**
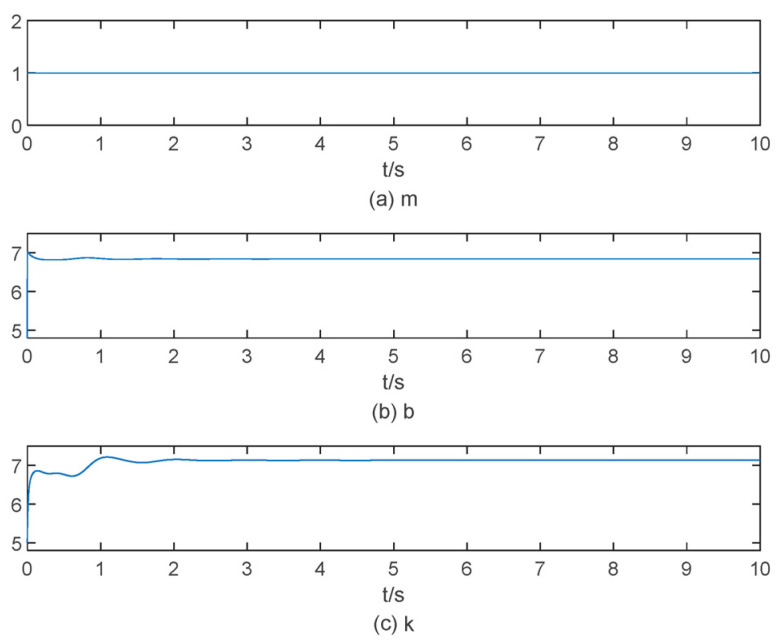
The change process of the impedance parameter when the object stiffness is 50 N/m.

When the object stiffness is increased to 50 N/m and the desired position signal $x_{d}$ is set to 0.2 m, the corresponding simulation results are illustrated in Figure 11 and Figure 12. In Figure 11, the overshoot is more pronounced compared to the other two simulations, with a settling time of approximately 4 s. In Figure 12, the impedance parameters stabilize at b=6.9 and k=7 within approximately 4 s. The force steady-state error values are presented in Table 2.

From the analysis of the simulation results, there is no significant difference in the stabilization time for both the system output position and force. The steady-state error converges to zero within approximately 4 s. Additionally, the adjustment time for the impedance parameters shows no significant variation, with the adjustment process concluding at around 4 s. It is observed that the impedance parameter adjustment concludes simultaneously with the system achieving stable tracking, aligning with the theoretical derivation presented in Section 3.4. The results from the variable-parameter impedance control simulation demonstrate that this method can self-regulate impedance parameters, reducing the force steady-state error to zero. However, during the self-regulation process, a significant overshoot in the output response is observed, which increases as the stiffness of the object increases. Requiring that the object’s maximum allowable force exceeds the overshoot measured in the experiment.

## 5. Experimental Verification

**Figure 13:**
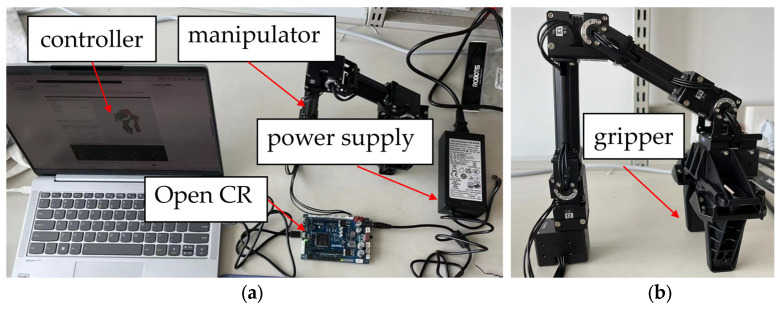
Manipulator gripping experiment platform. (**a**) The overall view picture of the experimental platform; (**b**) the side view of the manipulator.

To further assess the effectiveness of the proposed control method, an experimental platform was constructed, as shown in Figure 13a, The Open CR enabled communication between the controller (Lenovo Xiaoxin Air14 Plus laptop, Lenovo, Beijing, China) and the manipulator (Open Manipulator RM-X52, ROBOTIS Co. Ltd., Seoul, Republic of Korea). The controller operated on Ubuntu 18.04, and the manipulator was controlled via C++ programming in the ROS environment. The Open Manipulator RM-X52 consists of four joints and a gripper, each powered by an XM430-W350-T (ROBOTIS Co. Ltd., Seoul, Republic of Korea) servo. In our experiments, the angles of the first four joints are kept fixed at [0°, 90°, 0°, 0°], validating the control methods on the gripper. These experiments are conducted within the joint space. The open-source Dynamixel Workbench package was used to input control signals and feedback on the position and current signals.

The relevant parameters of the XM430-W350-T servo motor are listed in Table 3. In the experiment, the baud rate was set to 1,000,000 bps. The power supply delivers 12 V to the manipulator through the OpenCR (ROBOTIS Co. Ltd., Seoul, Republic of Korea) board. The moment of inertia of the motor was calculated using the mass and volume data from Table 3, resulting in a value of 0.00279 kg·m^2^. The minimum load was experimentally determined to be 0.0042 N·m. The parameters for the SMC in position control are set as Λ=14 and *k* = 5. Note that the parameter k here refers to the gain applied to the sign function in the sliding mode control law, intended to counteract disturbances in the system. This *k* should not be confused with the stiffness coefficient k used in the impedance controller.

**Figure 14:**
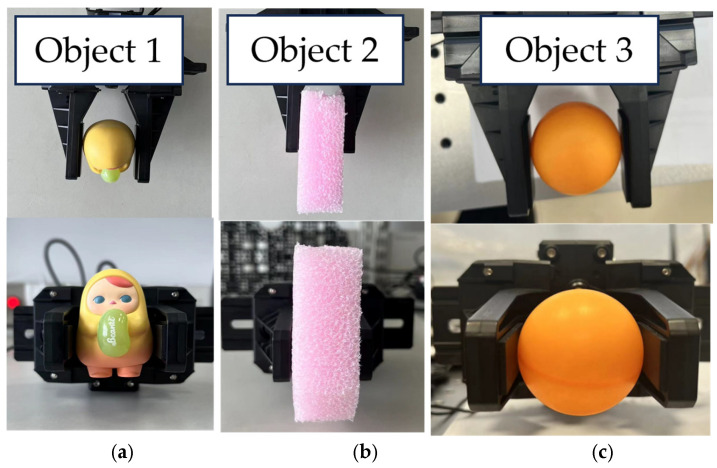
Top and main views of the manipulator during the grasping of object 1 (**a**), object 2 (**b**) and object 3 (**c**).

Experimental validation was conducted for the variable-parameter impedance control method. The objects used for gripping were a PVC blind box (object 1), a 2.5 cm EPE board (object 2), and a ping pong ball (object 3), as shown in Figure 14. Based on the tests, the stiffness values of the three objects, ranked from highest to lowest, are object 1, object 3, and object 2. The gripping process can be divided into three stages: (1) transitioning from the open state to initial contact with the object, (2) adjusting the gripping force, and (3) achieving a steady state.

The hidden layer of the RBF neural networks contains 5 nodes, with initial weights randomly assigned. The node centers are symmetrically spaced at equidistant values (−20, −10, 0, 10, 20), while the node widths are uniformly set to 40. The learning rate is 0.25, and the learning factor is 0.1.

**Figure 15:**
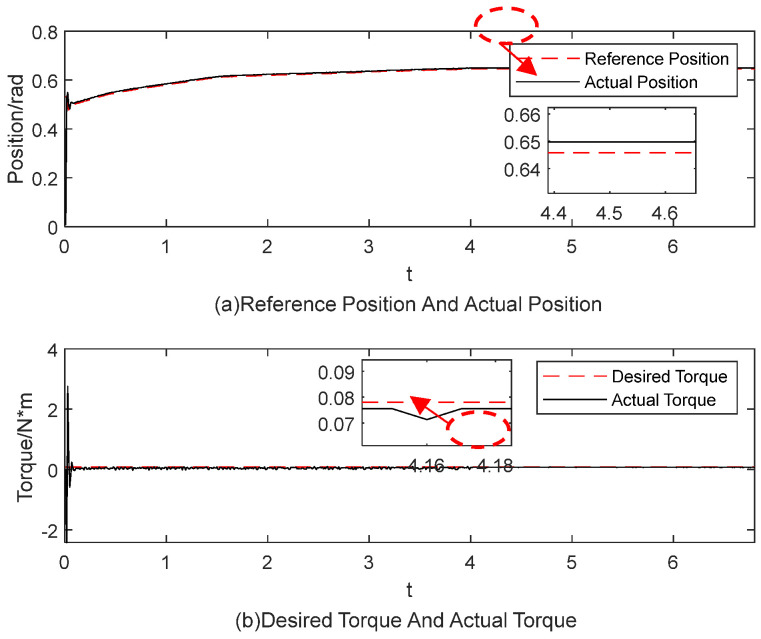
The experimental results for the variable-parameter impedance control method during the grasping of object 1.

**Figure 16:**
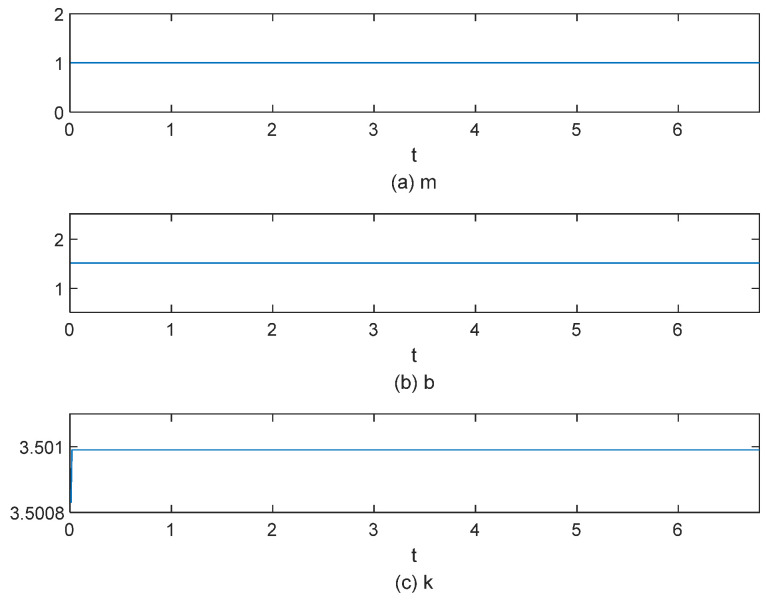
The impedance parameter variation process for grasping object 1.

**Figure 17:**
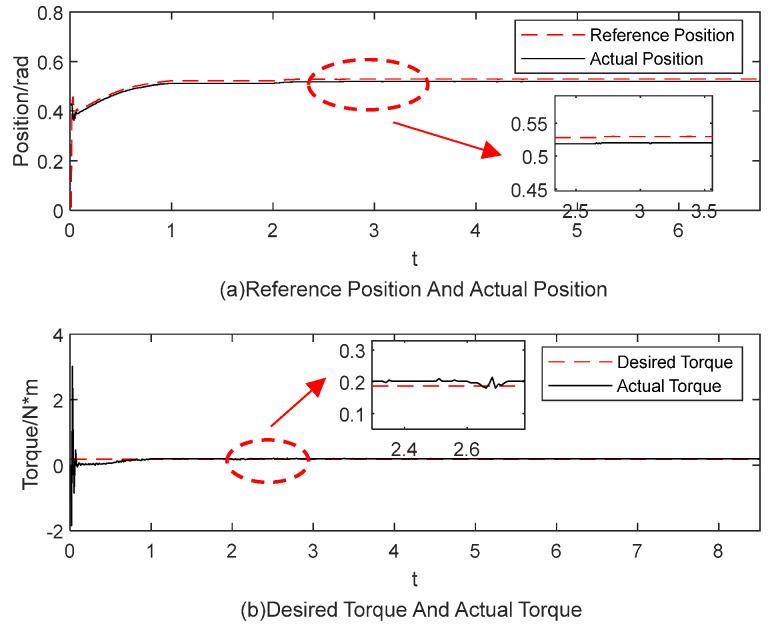
The experimental results for the variable-parameter impedance control method during the grasping of object 2.

**Figure 18:**
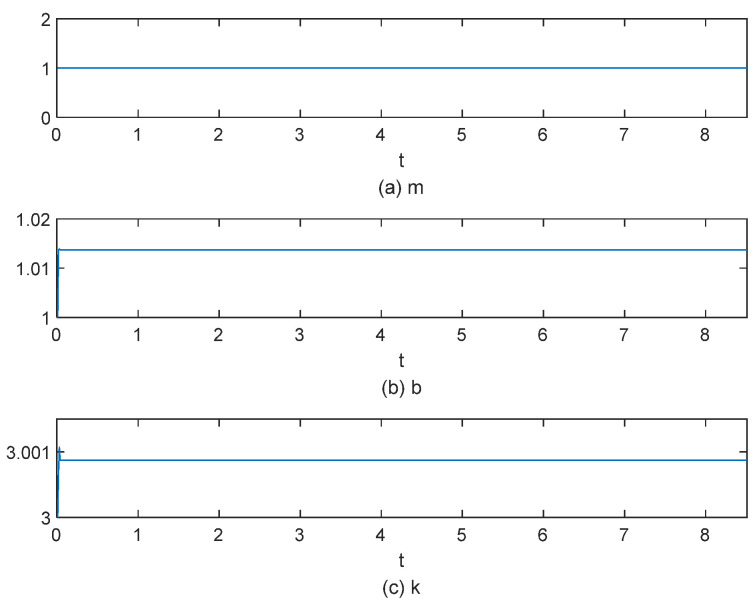
The impedance parameter variation process for grasping object 2.

**Figure 19:**
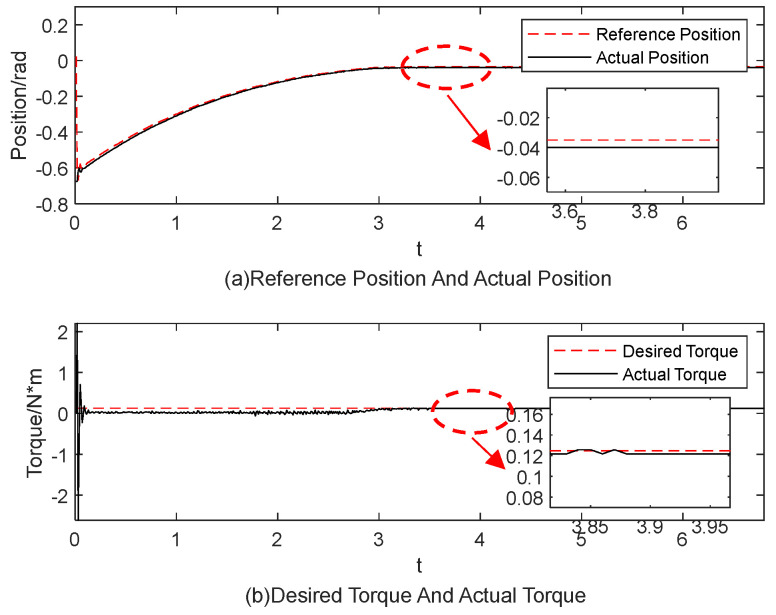
The experimental results for the variable-parameter impedance control method during the grasping of object 3.

**Figure 20:**
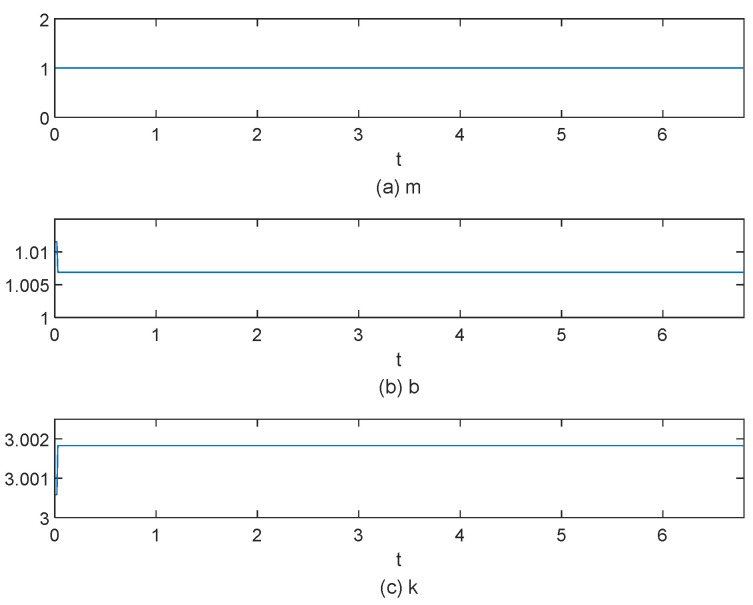
The impedance parameter variation process for grasping object 3.

Figure 15, Figure 16, Figure 17, Figure 18, Figure 19 and Figure 20 present the results from three experimental sets utilizing the variable-parameter impedance control method. Figure 15, Figure 17, and Figure 19 compare the actual and reference positions, as well as the actual and target forces of the gripper, during the grasping of objects 1, 2, and 3, respectively. The reference position is defined as the desired position adjusted by the position correction amount generated by the impedance controller. The actual position is the current position of the gripper, as measured by the absolute encoder. Both the reference and actual position data are provided in radians. Figure 16, Figure 18, and Figure 20 depict variations in impedance parameters across three experimental sets. The values of force steady-state errors are shown in Table 4. 

In Figure 15a, the red line denotes the reference position, while the blue line indicates the actual position. The gripper’s position signal tracks the reference position stably from the start. In Figure 15b, the red line denotes the desired torque, while the blue line indicates the actual torque, with both sets of data measured in N·m. The system enters a steady state of gripping around 4 s, with the torque steady-state error maintained at 0.0025 N·m. In Figure 16, the impedance parameters were adjusted and stabilized at *b* = 1.5 and *k* = 3.50098

In the experiment involving object 2, the system enters a steady state of gripping around 4 s, with the torque steady-state error maintained at 0.01 N·m. The position controller exhibits a rapid and stable response to the reference position signal, and the impedance parameters were adjusted and stabilized at *b* = 1.0135 and *k* = 3.00085.

In the final experiment, the position controller demonstrated consistent performance. The system entered a steady state of gripping around 3.5 s, with the torque steady-state error maintained at 0.01 N·m. The impedance parameters were adjusted and stabilized at *b* = 1.0054 and *k* = 3.00185. These findings highlight the robustness and adaptability of the control system across different experimental conditions, underscoring its potential for real-world applications in robotic manipulation tasks.

By comparing these three experiments, we can derive the following observations. The inner-loop position controller ensures a rapid response and stable tracking of input signals. During the initial phase, the motor generates significant torque to move the gripper from the open position to make contact with the object, which takes approximately 0.1 s. Subsequently, the system enters a phase of adjusting the gripping force, during which minor oscillations can be observed. The amplitude and duration of these oscillations vary depending on the stiffness of the object being gripped. Generally, the greater the object’s stiffness, the larger the amplitude and the longer the duration of the oscillations. Ultimately, upon entering the steady state, the force steady-state error remains within approximately 5%.

The experimental results confirm that the method proposed in this paper, in comparison to conventional fixed-parameter impedance control, eliminates the need for manual parameter tuning in grasping tasks and significantly enhances force control precision.

## 6. Conclusions

The conventional force/position hybrid control method is constrained by frequent controller switching, leading to high computational demands. Although impedance controllers reduce computational load, they lack adaptability to varying contact environments. Current approaches for the self-adaptation of impedance parameters often compromise position steady-state error or do not support online adjustments. To address these issues, this paper proposes a variable-parameter impedance control method based on the RBF neural networks and the gradient descent, for online impedance parameter adjustment. A sliding mode controller mitigates uncertainty in the manipulator, reducing position control error. We conducted simulations and experiments using a single-joint model in joint space. Simulations and experiments confirm the method’s efficacy in grasping objects with different stiffness levels, showing clear advantages over alternative approaches. 

In this study, we focused on a single-joint model, which does not account for the compliance of other joints. These factors will be addressed in future work to enhance the overall compliance of the manipulator and improve human-manipulator interaction.

## Figures and Tables

**Table 1:** Initial parameterization of the neural networks.

Number of Hidden Layer Nodes	Initial Weighting	Nodal Center	Node Width	Learning Rate	Momentum Factor
5	10	30	40	0.25	0.1

**Table 2:** Steady-state error value of simulation.

	**Simulation 1**	**Simulation 2**	**Simulation 3**
Desired force (N)	10	10	10
Force steady-state error (N)	0.003	0.0047	0.003

**Table 3:** The parameters of the XM430-W350-T servo.

	XM430-W350-T
MCU	ARM CORTEX-M3 (72 [MHz], 32 Bit)
Position Sensor	Contactless absolute encoder (12 Bit, 360 [°]) Maker: ams, Part No:AS5045
Motor	Coreless
Baud Rate	9600 [bps]~4.5 [Mbps]
Weight	82 [g]
Dimensions (W × H × D)	28.5 × 46.5 × 34 [mm]
Input Voltage	10.0~14.8 [V] (Recommended: 12.0 [V])

**Table 4:** Steady-state error value of the experiment.

	Experiment 1	Experiment 2	Experiment 3
Desired torque (N·m)	0.078	0.1872	0.1248
Force steady-state error (N·m)	0.0025	0.01	0.003

## Data Availability

The data that support the findings of this study are available from the authors upon reasonable request.
